# Whole-Genome Sequence of the Cheese Isolate *Lactobacillus rennini* ACA-DC 565

**DOI:** 10.1128/genomeA.01579-16

**Published:** 2017-02-02

**Authors:** Maria Kazou, Voula Alexandraki, Bruno Pot, Effie Tsakalidou, Konstantinos Papadimitriou

**Affiliations:** a Laboratory of Dairy Research, Department of Food Science and Human Nutrition, Agricultural University of Athens, Athens, Greece; b Research Group of Industrial Microbiology and Food Biotechnology (IMDO), Vrije Universiteit Brussel, Brussels, Belgium

## Abstract

In this study, we present the first complete genome sequence of *Lactobacillus rennini* ACA-DC 565, a strain isolated from a traditional Greek overripened Kopanisti cheese called Mana. Although the species has been associated with cheese spoilage, the strain ACA-DC 565 may contribute to the intense organoleptic characteristics of Mana cheese.

## GENOME ANNOUNCEMENT

*Lactobacillus rennini* is a Gram-positive homofermentative lactic acid bacterium belonging to the *Lactobacillus coryniformis* clade (1). *L. rennini* was originally found in spoiled rennet that, when used, produced a cheese with defects (2, 3). Strain ACA-DC 565 was isolated from a 2-year-old traditional Greek Kopanisti cheese (4). This overmature type of Kopanisti cheese, referred to as Mana, is used as an inoculum for the back-slopping production of Kopanisti cheese (5). *L. rennini* was the sole isolated microbial species in Mana producing alcohols and carbonyl compounds as major volatile compounds, most probably via the secondary catabolism of amino acids (4, 6). Mana cheese has an intense salty and a distinct piquant flavor (5), and strain ACA-DC 565 may contribute to the strong organoleptic characteristics of this cheese.

The ACA-DC 565 genome was sequenced on the Illumina HiSeq 2000 platform at the Beijing Genomics Institute (BGI Co. Ltd., Hong Kong) using three paired-end libraries with insert sizes of 500 bp, 2,000 bp, and 6,000 bp. Genome size was estimated by k-mer analysis. SOAPdenovo version 2.04 was employed to assemble the reads after filtering, and the resulting contigs were placed into superscaffolds (7, 8). The reads located in gaps were closed using local assembly and PCR gap closure. The final step of error correction was performed with the SOAPaligner/soap2 software. The assembly resulted in one circular chromosome of 2,350,601 bp, with a G+C content of 40.7% and one incomplete plasmid, which is still under sequencing (data not shown). Whole-genome optical mapping of strain ACA-DC 565 was used to validate the hybrid assembly (9). The map was generated at Microbion SRL (Verona, Italy), and the alignment between the assembled genome and an *Afl*II optical map was created with the Argus Optical mapping system (OpGen Technologies, Inc., Madison, WI, USA).

Genome annotation was performed with RAST version 2.0 (10). Genes were predicted combining the results of Prodigal (11), MetaGeneAnnotator (12), and FGENESB (13), while putative pseudogenes were identified with GenePRIMP (14). Finally, the Artemis tool (15) and the BLAST suite (16) were used for the manual curation of the genes. Concerning the functional annotation of the genome, proteins of strain ACA-DC 565 were searched against the Pfam database (17) and the Phobius Web server (18).

The ACA-DC 565 chromosome consists of 2,348 genes, including 2,166 protein-coding genes, 106 potential pseudogenes, 15 rRNAs, and 61 tRNA genes. The existence of potential pseudogenes may suggest genome decay to an extent, which is not unusual for food-related lactic acid bacteria. Furthermore, the chromosome of strain ACA-DC 565 contains 1,875 protein-coding genes with Pfam domains, 180 protein-coding genes with signal peptides, and 495 protein-coding genes with transmembrane helices.

Although *L. rennini* has been associated with cheese spoilage (2, 3), additional analysis of strain ACA-DC 565 may reveal technological properties that render it as a suitable starter or adjunct in Kopanisti cheese production.

### Accession number(s).

The chromosomal sequence of *L. rennini* ACA-DC 565 is deposited at the European Nucleotide Archive (ENA) under the accession number LT634362.
